# Supplementary material for: Development of algorithms for identifying patients with Crohn’s disease in the Japanese health insurance claims database
Source: PLoS One. 2021 Oct 13;16(10):e0258537. doi: 10.1371/journal.pone.0258537 (PMC8513890; doi:10.1371/journal.pone.0258537)
Supplement: S1 File — (DOCX) [file pone.0258537.s004.docx]

Investigation of risk factors for onset and relapse of Crohn's disease based on insurance claims database.

Vice Director and Associate Professor Taku Kobayashi

Affiliation: Kitasato Research Institute Hospital, Kitasato University

Department: Center for Advanced IBD Research and Treatment

Address 5-9-1 Shirogane, Minato-ku, Tokyo

Phone 03-3444-6161

Fax: 03-3448-0553

E-mail drkobataku@gmail.com

1

October 9, 2020 Plan, Version 3

1. Objectives of clinical research

In this study, target patients will be extracted from the claims database and their clinical information will be extracted. Examine risk factors for Crohn's disease and relapse. To assess the validity of the inclusion criteria, a medical record review will be performed on some of the extracted cases to evaluate the positive or negative predictive value of the inclusion criteria.

2. Patients

2.1 Inclusion criteria

Extract the following patients from the database held by JMDC Corporation.

① Patients diagnosed with CD using ICD-10 coding of K50 (Appendix 1).

② Patients diagnosed with CD using the ICD-10 coding of K50 (Appendix 1).

In addition to (1) and (2) above, patients who, within ±12 months from the date of diagnosis, have prescribe code or procedure code listed in the national guidelines for Crohn's disease. (Reference 1)

③Patients who do not meet the above criteria.

2.2 Exclusion Criteria

There are no exclusion criteria for this study.

 2.3 Patients for inclusion criteria validity assessment

Patients who visited Kitasato University Kitasato Institute Hospital from May 1, 2012, to March 31, 2019, and meet the following criteria will be eligible.

(1) 100 cases each of patients who meet the inclusion criteria ① and ②

(2) 200 cases of patients who meet ③

3. Informed consent

Since this research uses receipt data and medical records, no written explanation or consent will be obtained by the "Ethical Guidelines for Medical and Health Research Involving Human Subjects”. By this guideline, informed consent was obtained in the form of opt-put on the web-site. In this way, patients or surrogates patients who refuse to participate in the study will be removed from the analysis and immediately disposed of.

4. Methods of clinical research

4.1 The designs of clinical research

A case-control study conducted with JMDC co, Ltd,.

4.2 Procedure

This study was conducted in collaboration with JMDC Corporation, a company whose main business is to provide medical statistics data services by maintaining receipts (inpatient, outpatient, and dispensing) and medical examination data from multiple health insurance associations.

The data has been accumulated since 2005, and the cumulative population is approximately 5.6 million as of June 2018. About 4,000 target cases are extracted from the database (anonymized processed information) held by JMDC, using extraction criteria. JMDC will construct analysis data from the clinical information, and conduct statistical analysis of the onset and relapse factors of Crohn's disease from the constructed analysis data.

A validity evaluation of the inclusion criteria will be also conducted. JMDC, Inc. will extract the 400 cases of patients in Kitasato University Kitasato Institute hospital to be used for the validity evaluation. JMDC will extract a total of 400 patients who meet and do not meet the criteria from the receipts held by the hospital, and anonymize them by assigning a research number to the research subject so that no specific individual can be identified at this facility (Correspondence table provided by Yuki Watanabe, Personal Information Manager). The corresponding table will be kept in a locked cabinet by the principal investigator, and the information will not be taken outside the facility. Cases will be selected from the anonymized data according to the inclusion criteria, and random sampling will be conducted. A medical chart review to determine whether the cases had Crohn's disease was conducted by a research physician. Based on the results of the medical record review, the validity of the inclusion criteria will be assessed by calculating the positive/negative predictive value, sensitivity, and specificity.

4.3 Data Management

The analysis data extracted from the hospital's receipt information will be stored appropriately in JMDC's information system in accordance with JMDC's information security rules in compliance with ISMS (Contact: Takayoshi Nagahama). The correspondence chart will be strictly managed by Yuki Watanabe, the personal information manager in the hospital.

4.4 Case Registration Method

Extract the following information about the target case.

1) Date of diagnosis of Crohn's disease

2) Patient background (gender, age, height, weight, medical history, family history, current medications, and (Crohn's disease, time of onset (disease duration), disease type)

3) Presence or absence of complications such as anal lesions, stenosis, fistula, skin, arthritis, etc.

4) Lifestyle factors such as alcohol consumption and smoking habits before the onset of Crohn's disease

5) Lifestyle factors such as alcohol consumption and smoking habits after the onset of Crohn's disease

6) Therapeutic drugs (mesalazine, steroids,

immunomodulators, biologics) (Drugs listed in the diagnostic criteria and treatment guidelines for Crohn's disease*1 such as

(7) NSAIDs, antibacterials, statins, oral contraceptives, and other drugs related to the onset and relapse of Crohn's disease. History of use of the drug to be given

8) Availability and date of surgery

9) Hospitalization and date

10) Presence and date of malignancy in and outside the intestinal tract 5. evaluation items

 5.1 Primary endpoints

Evaluation of risk factors associated with the development of Crohn's disease and relapse.

5.2 Secondary endpoints

The validity of the extraction conditions (positive predictive value, negative predictive value, sensitivity, specificity).Report on deviations from the implementation plan

The principal investigator must obtain prior approval from the Kitasato Research Institute Hospital Research Ethics Committee before implementing the clinical research protocol. Besides, it is prohibited to deviate from or change the in the event of any deviation from the clinical research protocol, the principal investigator shall notify the investigator of the deviation. (2) The researcher shall record all of the following information, along with the reasons for it, and report it to the Research Ethics Committee.

7. discontinuation and dropout criteria

 7.1 Discontinuation of enrolled patients

There are no dropout criteria for this study, but since it is conducted using an opt-out

Patient data that have been requested not to participate in the study by opting out will be used for data extraction.

Excluded at the time of data extraction. Requests made after data extraction will be excluded from published materials.

7.2 Stopping the research itself

When the principal investigator decides that the research should be discontinued.

In the above cases, report to the hospital director and the Kitasato Research Institute Hospital Research Ethics Committee.

8. expected adverse events and emergency reporting methods In this study, we extracted and analyzed anonymized information from the medical fee database.

This is a study without medical intervention. No expected adverse events are expected.

9. analysis method

(1) Analysis target

All enrolled patients will be included in the analysis.

(2) Statistical analysis

Perform statistical analysis using the following methods, as appropriate.

Contingency table analysis: χ-square test, Fisher's exact test Comparison of two unpaired groups: unpaired t-test, Mann-Whitney U test.

Correspondence between two groups: Correspondence t-test, Wilcoxon sign rank test.

Survival time analysis: Cox regression analysis, Log-rank test Multivariate analysis: Logistic Regression Analysis

(indicating an approximate quote or vague suggestion) or something.

(3) Person responsible for analysis

Taku Kobayashi, Deputy Director, Center for Advanced Inflammatory

7

Bowel Disease, Kitasato Institute Hospital, Kitasato University

(4) Evaluation of analysis results

The results of the analysis will be published after evaluation by the Center for Advanced Therapy of Inflammatory Bowel Disease.

10. expected research period

Case accumulation period: After approval by the director of the institution - September 30, 2020 (A.D.)

Validity evaluation period: After approval by the director of the institution - September 30, 2020 (A.D.)

Case analysis period: After approval by the director of the institution - March 31, 2021 (A.D.)

The number of cases to be studied and the basis for setting the number (1) Number of cases to be studied The expected number of cases is the total number of Crohn's disease cases recorded in the JMDC database target.

The number of cases required for validity assessment is 100 cases for criteria (1) and (2), and 200 cases for (3). (2) The basis for setting if the positive predictive value is set at 80% (95% confidence interval ± 0.1), the sample size required for validity assessment is 100 cases or criteria (1) and (2), and 200 cases for (3).

Location and responsible person for storage of case records Center for Advanced Therapy of Inflammatory Bowel Disease, Kitasato Institute Hospital, Kitasato University (Storage Manager) Yuki Watanabe (Storage method) Manage after confirming that no personal information is included.8

Principal Investigators and Assigned Researchers

(1) Principal Investigator

Center for Advanced Therapy of Inflammatory Bowel Disease, Kitasato Institute Hospital, Kitasato University

Address: 5-9-1 Shirokane, Minato-ku, Tokyo 108-8642, Japan Phone: 03-3444-6161

Taku Kobayashi (Inflammatory Bowel Disease Advanced Treatment Center)

(2) Research subcontractors

Hiromu Morikubo (Gastroenterology)

Tomohiro Fukuda (Gastroenterology)

Yuki Watanabe (Advanced Treatment Center for Inflammatory Bowel Disease)

(3) Collaborative research organization

JMDC Corporation Responsible for: Yoshitaka Nagahama, Norihisa Kodaira Job Description: Extract data from receipt data and medical chart information (electronic medical records) and construct analytical data. Statistical analysis methods and interpretation of results.

14. termination of clinical research

At the end of the clinical research, the principal investigator shall promptly submit a report on the completion of the clinical research to the Research Ethics Committee of Kitasato Research Institute Hospital.

15. Consideration for the human rights of the subject.

Those involved in the implementation of this research will give due consideration to the protection of the personal information of the subjects. When publishing the results of clinical research, information that can identify the subject should not be included, and the subject's data obtained from clinical research should not be used for any purpose other than the purpose of clinical research.

The data shall be stored in a lockable vault.

16. research costs and compensation

  Research Costs and Cost Sharing

This study was conducted in collaboration with JMDC, and JMDC provided the necessary funds for the study and The IBD Center will be provided with 2 million yen as research expenses. This research was funded by JMDC, Inc. This study was funded by our collaborator JMDC, Inc.

However, research funds are subject to review by the Kitasato University Conflict of Interest Review Committee. It is properly managed, and the researcher must use his or her professional judgment for personal gain. There is no such thing as bending the rules. 16.2 Compensation for Health Damage. This research is a case-control study using receipt information and medical information not applicable.

17. ethical matters

17.1 Compliance with Ethical Guidelines and Declaration of Helsinki

This research will be conducted in compliance with the latest "Declaration of Helsinki" and "Ethical Guidelines for Medical Research Involving Human Subjects," etc.

17.2 Review and Approval by the Ethics Committee

 This clinical research will be started after approval by the Research Ethics Committee and permission by the head of the institution.

17.3 Informed Consent

 Since this research uses receipt data and medical records, no written explanation or consent will be obtained in accordance with the "Ethical Guidelines for Medical Research Involving Human Subjects. In order to disclose the "matters that should be disclosed regarding the conduct of the research in the event that informed consent is not obtained" as indicated in the guidelines and to give the subject or a substitute the opportunity to refuse to participate in the research, materials regarding opt-out will be posted, and the data of subjects who refuse to participate in the research will be deleted from the analysis and immediately destroyed. The data of subjects who refuse to participate in the study will be removed from the analysis and immediately destroyed.

17.4 Protection of Personal Information and Disclosure of Data

JMDC Inc. will extract cases from the hospital's receipt information, anonymize the data so that no specific individuals can be identified, and construct the data, but the corresponding tables will be kept in strict confidence at the hospital. JMDC will delete the constructed data as soon as the study is completed. JMDC will delete the data as soon as the study is completed. The results of this study will be published in academic journals, but no personally identifiable information will be disclosed. The data obtained in this study will not be used for any other purpose than this study.

18. Publication of research results and attribution of intellectual property rights

Research results will be used as materials for conference presentations, academic information provision, etc. The research results will be presented at academic conferences and published in academic journals and databases within one year of the completion of the research.

19. Changes to the implementation plan, etc.

 Any changes (revisions) to the research protocol must be approved by the Research Ethics Committee in advance.

References

1. Ministry of Health, Labour and Welfare, "Survey and Research on Intractable Inflammatory Bowel Ginger" (Suzuki Group)

Diagnostic criteria and treatment guidelines for ulcerative colitis and Crohn's disease, revised in 2008

21. appendix

 Appendix 1 ICD-10 codes used for extraction criteria K50.0 Crohn's disease of small intestine

 Crohn's disease of the ileum

 Crohn's disease of the jejunum

 Crohn's disease of the duodenum

 Crohn's disease of the small intestine

K50.1 Crohn's disease of large intestine

 Crohn's disease of the colon

 Crohn's disease of the appendix

 Crohn's disease of the rectum

K50.8 Other Crohn's disease

 Gastric Crohn's disease

 Gastroduodenal Crohn's Disease

 Crohn's disease of the anus

 Crohn's disease of small and large intestine K50.9 Crohn's disease, details unknown.   Crohn's disease

 Crohn's Juvenile Arthritis

 Steroid-Dependent Crohn's Disease
